# Pathogenesis of Hand-Foot Syndrome induced by PEG-modified liposomal Doxorubicin

**DOI:** 10.1007/s13577-012-0057-0

**Published:** 2013-02-06

**Authors:** Noriyuki Yokomichi, Teruaki Nagasawa, Ariella Coler-Reilly, Hiroyuki Suzuki, Yoshiki Kubota, Ryosuke Yoshioka, Akiko Tozawa, Nao Suzuki, Yoko Yamaguchi

**Affiliations:** 1 Department of Obstetrics and Gynecology, St. Marianna University School of Medicine, 2-16-1 Sugao, Miyamae, Kawasaki, Kanagawa 216-8511 Japan; 2 NANOEGG® Research Laboratories Inc, 2-16-1 Sugao, Miyamae, Kawasaki, Kanagawa 216-8512 Japan; 3 Institute of Medical Science, St. Marianna University School of Medicine, 2-16-1 Sugao, Miyamae, Kawasaki, Kanagawa 216-8512 Japan

**Keywords:** Hand-Foot Syndrome (HFS), Palmar-plantar erythrodysesthesia (PPE), Pegylated liposomal doxorubicin, Drug delivery system (DDS), Reactive oxygen species (ROS)

## Abstract

PEGL-DOX is an excellent treatment for recurrent ovarian cancer that rarely causes side-effects like cardiotoxicity or hair loss, but frequently results in Hand-Foot Syndrome (HFS). In severe cases, it can become necessary to reduce the PEGL-DOX concentration or the duration of the drug therapy, sometimes making it difficult to continue treatment. In this study, we prepared an animal model to compare the effects of DOX versus PEGL-DOX, and we noticed that only treatment with PEGL-DOX resulted in HFS, which led us to conclude that extravasation due to long-term circulation was one of the causes of HFS. In addition, we were able to show that the primary factor leading to the skin-specific outbreaks in the extremities was the appearance of reactive oxygen species (ROS) due to interactions between DOX and the metallic Cu(II) ions abundant in skin tissue. ROS directly disturb the surrounding tissue and simultaneously induce keratinocyte-specific apoptosis. Keratinocytes express the thermoreceptor TRPM2, which is thought to be able to detect ROS and stimulate the release of chemokines (IL-8, GRO, Fractalkine), which induce directed chemotaxis in neutrophils and other blood cells. Those cells and the keratinocytes then undergo apoptosis and simultaneously release IL-1β, IL-1α, and IL-6, which brings about an inflammatory state. In the future, we plan to develop preventative as well as therapeutic treatments by trapping the ROS.

## Introduction

Standard cancer treatments currently include surgery, radiation therapy, and chemotherapies such as anticancer drugs; however, there are advantages and disadvantages to each treatment. The specific course of treatment is chosen based on the cancer’s rate of progression, but in most cases, the standard of care is to choose a chemotherapeutic anticancer drug. Hair loss, pancytopenia, and nausea/vomiting are the side-effects most typically seen with anticancer drug treatment, but there have also been serious adverse reactions in the skin. These reactions cause intense pain in the hands and feet, and the phenomenon is known as Hand-Foot Syndrome (HFS) or Palmar-Plantar Erythrodysesthesia (PPE) [1]. In cases where HFS is severe, patients experience difficulty walking and lose the ability to hold objects in the hands, everyday life becomes significantly impaired, and it may become difficult to continue the cancer treatment.

Anticancer drugs that have been found to frequently cause outbreaks of HFS include: fluoropyrimidines like capecitabine, which is used to treat colorectal cancer and breast cancer; anthracycline, which is used against malignant solid tumors; a PEG-modified liposomal doxorubicin formulation (PEGL-DOX), which is used against recurrent ovarian cancer [2]; docetaxel, which is used against a wide variety of cancers; and sorafenib or sunitinib, which are molecular target drugs used against kidney cancer. The frequencies with which certain drugs can induce HFS are ranked as follows: PEGL-DOX as the highest at approximately 78 %, next is capecitabine at 51–78 %, and finally sorafenib at about 55 % [3].

Anticancer drugs are made to cause cellular malfunctions, but the particular reasons for the occurrence of side effects like pain in the hands and feet, reddening and cracking of the skin, numbness, and erythema (red spots) are not understood in much detail. For that reason, there are currently no effective treatments, and the standard practice is to recommend moisturizer, or topical steroids in severe cases.

PEGL-DOX is exceedingly effective in treating recurrent ovarian cancer without causing side effects like cardiotoxicity, neutropenia, anemia, alopecia, or nausea/vomiting. However, because of the frequency with which HFS occurs, there is a high risk that the patient’s quality of life will severely decline. With regards to insuring the completion of medical treatment, it is extremely important to establish precautionary measures. Therefore, in order to elucidate the mechanism of HFS outbreaks, we carried out in vivo experiments to analyze skin histology in the extremities, cytokine analyses, and in vitro experiments in skin cells.

## Materials and methods

### Chemicals

Doxorubicin (DOX, generic for Adriamycin) was obtained from Nippon Kayaku (Tokyo, Japan). In order to make PEGL-DOX (trade name Doxil^®^), a PEG-modified liposomal formulation of DOX, lecithin and polyoxyethylenated lecithin were obtained from NOF (Tokyo, Japan). Methanol, chloroform, copper chloride, and Mayer’s Hematoxylin and Eosin were purchased from Wako Pure Chemical Industries (Osaka, Japan). Fluorescein sodium salt was purchased for the fluorescent dye experiments from Sigma-Aldrich (St. Louis, MO, USA). DeadEnd™ Fluorometric TUNEL System was purchased for TUNEL staining from Promega (Madison, WI, USA).

### Animals

Six-week-old female SD rats and hairless rats were purchased from Japan SLC (Shizuoka, Japan), and were used for experiments after 1 week of rest. The rats were raised in independent cages in 50–60 % humidity, 23 ± 1 °C environment with light from 0630 to 1830 hours (12-h light–dark cycle) and were given ad libitum access to food and water. All animal experiments were carried out in accordance with the Guidelines for Animal Experimentation of St. Marianna University School of Medicine.

### Cell culture

From in vitro experiment, pathogenesis of HFS was expected to be a leakage of doxorubicin from peripheral vascular in the dermal layer in skin. As it was assumed that the cell in skin should be directly affected by doxorubicin alone, all in vitro experiments have been performed with doxorubicin alone.

HaCaT cell lines derived from human keratinocytes were received from Dr. Masamitsu Ichihashi of the Kobe University Graduate School of Medicine, Department of Dermatology. Normal Human Dermal Fibroblasts (NHDF cells) were purchased from KURABO Industries. HaCaT were cultured using Dulbecco’s Modified Eagle Medium (DMEM; Life Technologies, Carlsbad, CA, USA), and NHDF were cultured using DMEM supplemented with GlutaMAX™ Supplement I (Life Technologies, Carlsbad, CA, USA). The media were supplemented with 10 % fetal bovine serum, 100 U of penicillin, and 100 U of streptomycin. The cells were cultured at 5 % CO_2_ and 37 °C.

### Preparation of the PEGL-DOX

PEGL-DOX was produced from raw materials in the laboratory according to the Sadzuka method [4], but the steps involving filtration to equalize the particle sizes and the subsequent confirmation of the average particle size were omitted. l-α-distearoylphosphatidyl-dl-glycerol (DSPG, a PEG-modified lecithin) and lecithin were dissolved in a 1:4 methanol:chloroform solution and formed a thin film on the surface of the flask after the solvent was removed using a rotary evaporator in a 35 °C hot water bath. Aqueous DOX solution and sorbitol/lactic-acid solution was then added to the flask, and it was stirred for 15 min in a 60 °C hot water bath. After 3 min of sonication, the final product was a 0.2 % weight/volume PEGL-DOX solution. Physicochemical properties of PEGL-DOX were confirmed the following methods: (1) observation of a flow birefringence under a polarizing plate, and (2) semi-transparent without turbidity because of emulsified turbid appearance of liposome without modified PEG.

### Preparation of the HFS animal models

In order to determine whether the liposome formulation of doxorubicin developed from HFS onset, we have carried out comparative experiments with doxorubicin alone. To prepare the HFS animal models, PEGL-DOX and/or DOX (at 10 and 5 mg/kg, respectively) was administered to SD rats via the tail veins once every 3 days for 10 days. The limbs were visually inspected and photographed 10 days later. Afterwards, skin tissue samples from the hind limbs were collected, fixed in formalin, and embedded in paraffin. In non-clinical studies reported by dealers in DOXIL^®^, they did not find onset HFS for each additive of the PEGL-DOX preparation. Thus, in the present study, administration of the experiment in vivo was not performed for each additive to confirm the onset of HFS.

### Tissue Staining

The formalin-fixed paraffin-embedded skin tissue was sliced into 4 μm sections, deparaffinized, rehydrated, H&E stained, and then observed under an optical microscope. Picrosirius Red staining was applied in order to observe the state of the dermal collagen fibers and visualized under a polarized light microscope. TUNEL staining with the DeadEnd™ Fluorometric TUNEL System was used to detect apoptosis and visualized under a fluorescence microscope (BIOZERO; KEYENCE, Osaka, Japan).

### Measurement of cytokine expression in vivo and in vitro

Inflammatory cytokines and chemokines were measured in order to investigate the origins of HFS. After the application of the PEGL-DOX treatment, the hind-leg skin tissue was collected and used to measure in vivo expression levels. First the tissue was homogenized, then the samples were centrifuged, and finally the supernatant was analyzed using the Rat Cytokine Antibody Array (RayBiotech, North Metro-Atlanta, GA, USA). In addition, in vitro experiments were conducted in order to clarify the influence of DOX on skin cells, specifically epithelial cells. HaCaT cells were cultured in media supplemented with DOX and CuCl_2_, and the inflammatory cytokines were measured using the Human Cytokine Antibody Array (RayBiotech). IL-8, GRO, and Fractalkine chemokines of the CXC family, which corresponds to the CINC3 rat chemokine family were quantified using the Luminex200 system (Millipore, Billerica, MA, USA). Similar experiments were also carried out using NHDF.

### Creation of a visualizable model of a PEG-modified liposomal drug using fluorescein

In order to investigate the phenomenon by which HFS develops selectively in the limbs rather than throughout the whole body, fluorescein (FS) was used to create an easily visualizable model of a PEG-modified liposomal drug. The PEG-modified liposomal fluorescein (PEGL-FS) drug was prepared exactly as the PEGL-DOX drug was prepared. FS or PEGL-FS was administered to hairless rats via the tail vein, and whole-body FS was visualized under a long-wavelength ultraviolet lamp (UVGL-58 Handheld UV Lamp; UVP, Upland, CA, USA). Observations commenced immediately after drug administration, and photographs were taken periodically. Skin samples were collected from the soles of the hind-paws at 1, 7, and 24 h after drug administration. The OCT compound-embedded tissue was cut into 10-μm frozen sections and observed under a fluorescence microscope.

### Measurement of DOX toxicity in vitro

**Fig. 1:**
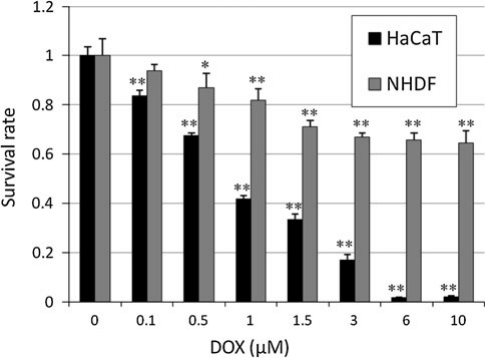
Survival rate of human skin cells following DOX treatments. HaCaT and NHDF cells were cultured with DOX for 24 h before counting. The values are presented as mean ± SD (*n* = 3). Significantly different from control: *p < 0.05, **p < 0.01

HaCaT and NHDF cell cultures were used to evaluate the toxicity of DOX in vivo. DOX was added at various concentrations (0.1–10 μM) to the media, and the percentage of viable cells was measured 24 h later using Cell Counting Kit-8 (CCK8; Dojindo Laboratories, Kumamoto, Japan). Results showed that 1.5 μM exhibited a moderate level of toxicity that was appropriate for the toxicity tests to follow (Fig. 1). To test the toxicity in the presence of copper ions, various concentrations of copper chloride (50 or 375 μM) were added to 1.5 μM DOX media. After 24 h of culturing, the survival rate was again measured using CCK8. Finally, to test the degree of inhibition of ROS by SODs, 100 μg/ml SOD (Sigma-Aldrich) was added to the medium, and 12 h later the survival rate was again measured.

### Statistical analysis

Dunnett’s and Tukey’s multiple comparison tests were used to analyze the results of in vitro experiments. The software used was R v.2.15.1 [5].

## Results

### Injections of PEGL-DOX yielded an HFS-like disease state

**Fig. 2:**
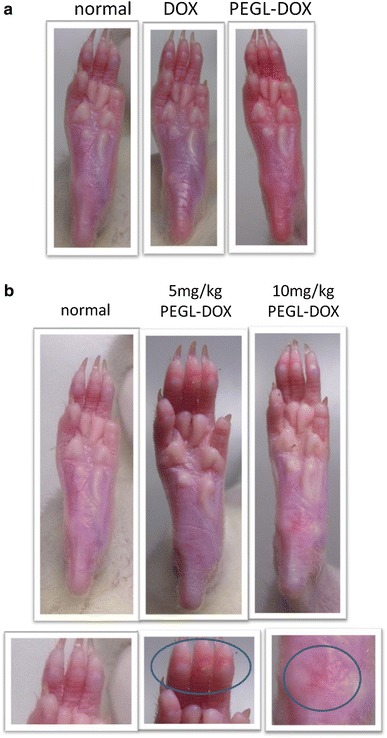
Rat paws (SD, female, 7 weeks) after intravenous injection of DOX or PEGL-DOX. **a** Appearance immediately after 10 mg/kg injection. **b** Appearance after multiple doses. Doses were administered once every 3 days, and photos were taken on the 10th day. *Blue circles* indicate particularly inflamed areas

Single or multiple doses of high-dose (10 mg/kg) or low-dose (5 mg/kg) DOX or PEGL-DOX were administered intravenously to SD female rats, whose limbs were then observed for signs of inflammation or redness. Changes in appearance were compared within single-dose or multiple-dose groups (Fig. 2a, b).

Within the single-dose group, immediately following PEGL-DOX administration, reddening was observed in the forepaws, hind-paws, ears, and at the tip of the nose; however, no such change was observed after DOX administration even after high-dose treatment (Fig. 2a). The redness that appeared was transient and disappeared after 5–10 min. Since the thickness of the rat limb skin is very thin, we can normally see the blood vessel through the skin. Because PEGL-DOX has a red color, observed transient redness after injection would correspond to the natural color of PEGL-DOX. Within the multiple-dose group, inflammation was observed after multiple low-dose PEGL-DOX treatments, and the change was even more striking after high-dose treatments (Fig. 2b). This observed state of inflammation, swelling, and dryness was judged to be similar enough to human HFS to conclude that HFS had indeed broken out in these rat limbs [6].

### Skin tissue staining revealed multiple adverse affects of high-dose PEGL-DOX

**Fig. 3:**
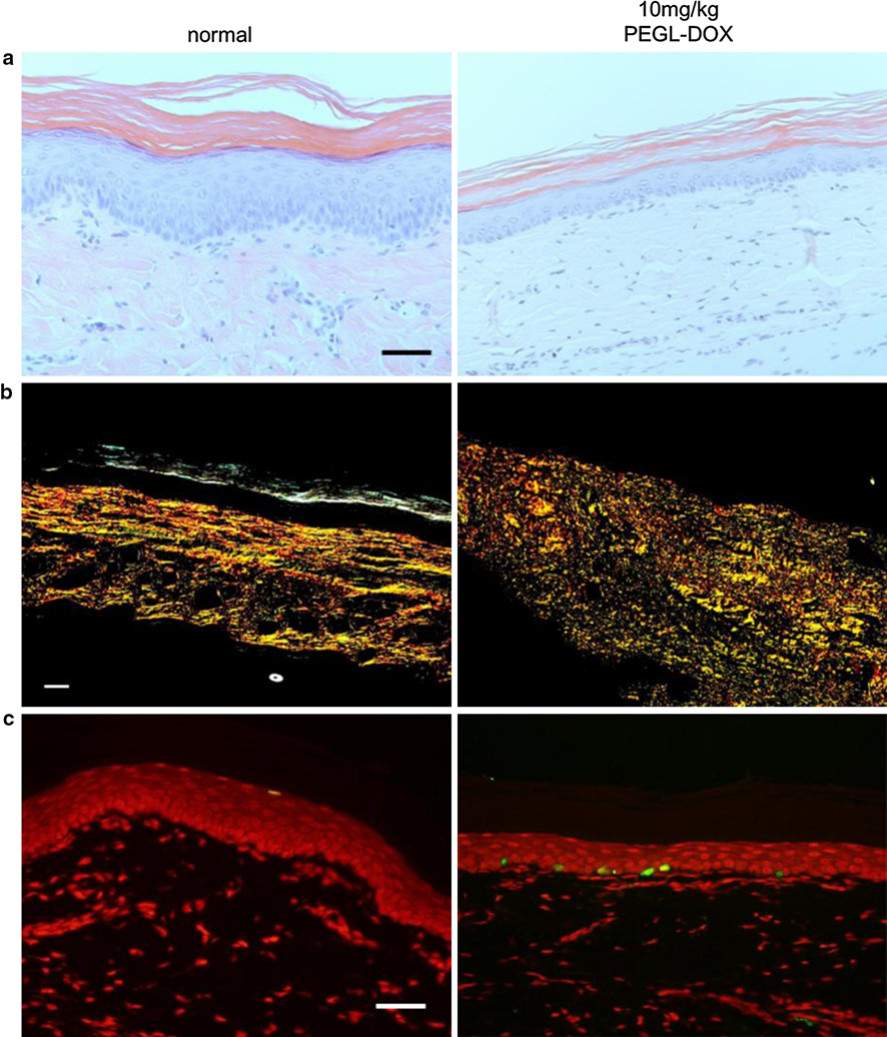
Tissue staining in rat (SD, female, 7 weeks) paw skin after 10 mg/kg PEGL-DOX injection. **a** H&E staining. Epidermal layer was thinned with respect to control (epidermal layer is shown in* blue* between the* pink* stratum corneum and* lighter blue* dermal layer). **b** Picrosirius red staining. Color of stain in order of decreasing strength and thickness of fibers: *red*, *yellow*, *green*. PEGL-DOX group displayed disarranged and broken collagen fibers. **c** TUNEL staining. *Red marks* nuclei, *green marks* apoptosis. Only basal cells show signs of apoptosis. (**a**–**c**)* scale bar* 50 μm

H&E staining clearly revealed the following effects of multiple doses of high-dose PEGL-DOX as compared to an untreated control group: a thinned or even absent granular layer, a decrease in the number of cells between the basal layer and the stratum spinosum, a rougher arrangement of cells, and a thinning of the epithelial layer (Fig. 3a). On the other hand, the dermal fibroblasts appeared relatively unaffected. Picrosirius red staining, which stains collagen fibers, revealed disarranged and broken collagen fibers in the multiple-dose PEGL-DOX group (Fig. 3b). TUNEL staining, which is a marker for apoptosis, showed that apoptosis was induced in basal epidermal cells in the PEGL-DOX group (Fig. 3c). In other words, the results of the TUNEL staining imply that HFS is related to apoptosis induced in epidermal cells.

### Antibody array showed increased expression of chemokines and inflammatory cytokines

**Fig. 4:**
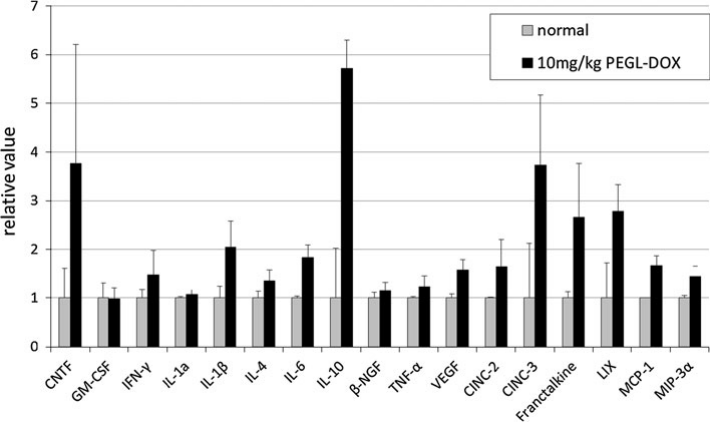
Rat Cytokine Antibody Array. Skin tissue from HFS-affected areas after multiple 10 mg/kg PEGL-DOX injections. Results were normalized to controls. *Graph* shows increased production of chemokines with respect to control. The values are presented as mean ± S.D

The proteins expressed in the regions of the rat skin tissue affected by the PEGL-DOX treatment were measured using an antibody array. Markedly increased expression of multiple proteins was confirmed: the chemokines CINC3 and Fractalkine, the IL-family-inhibitory IL-10, and inflammatory cytokines such as IL-1β and IL-6 (Fig. 4).

### PEGL-FS yielded more persistent fluorescence than unaltered FS in rat paws

**Fig. 5:**
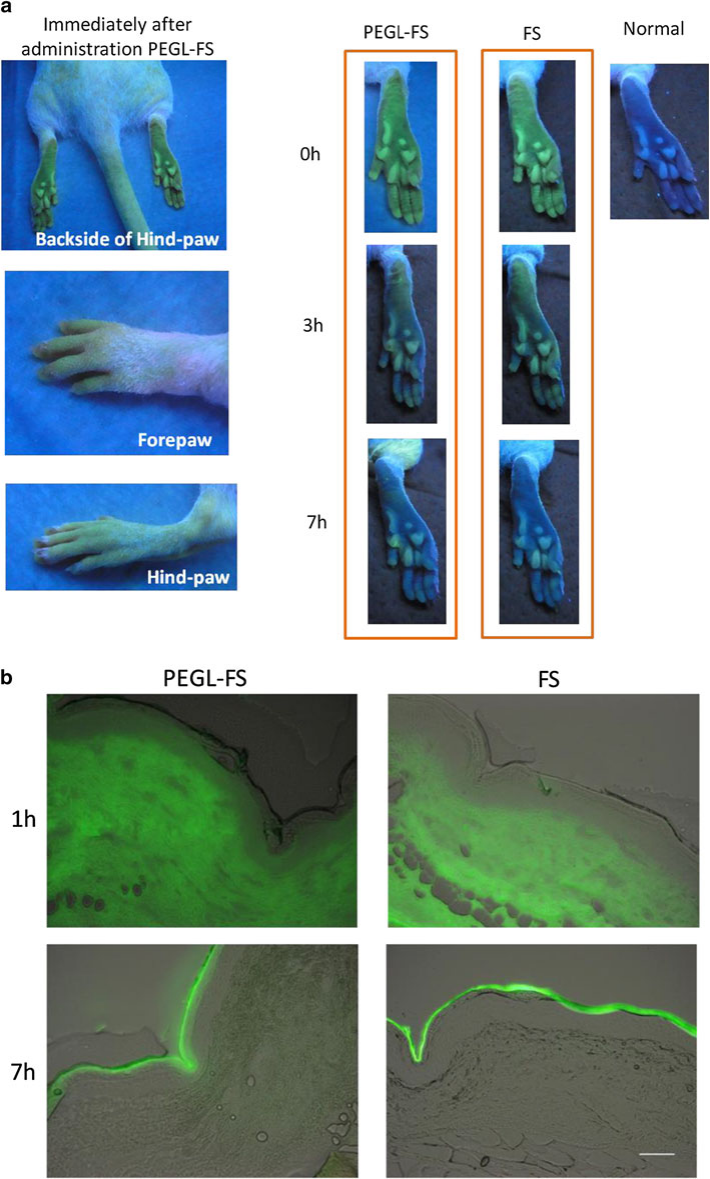
Comparison of photographs after intravenous injection of 36 mg/kg 1.5 ml PEGL-FS or FS in hairless rats (female, 7 weeks). **a** Photos cropped from whole-body visualization under long-wavelength UV lamp immediately after, 3 and 7 h after injection. PEGL-FS remained fluorescing in the paws 7 h post-injection. **b** Tissue sections cut 1 h and 7 h after injection. PEGL-FS diffused faster from the dermis through the epidermis to the stratum corneum, lingered in the dermis longer, and showed slightly higher fluorescence than FS. *Scale bar* (**b**) 100 μm

Immediately following administration of PEGL-FS or FS (unaltered fluorescein), very strong fluorescence was observed in the extremities. This fluorescence weakened over time but remained strong in the paws even after 3 h, and a small amount of fluorescence remained after 7 h in the PEGL-FS group (Fig. 5a).

Tissue sections at 1 h after treatment with either PEGL-FS or FS exhibited fluorescence over the entire dermal layer, indicating a high level of FS retention. It is assumed that the FS leaked out of the capillaries in the dermal layer (Fig. 5b). At this point, the PEGL-FS had already started to spread to the epidermis and exhibit fluorescence there. In sections at 7 h after treatment, the fluorescence had become concentrated at the upper stratum corneum, which suggested that the fluorescent dye had diffused from the dermis through the epithelium and arrived at the stratum corneum. In the PEGL-FS group, in contrast to the FS group, a small but noticeable amount of fluorescence remained in the dermis. In addition, the concentration of fluorescence in the stratum corneum appeared slightly higher in the PEGL-FS group than the FS group.

### DOX and Cu(II) ions increased production of chemokines and cytokines and lowered cell survival rates, which were rescued by SOD

**Fig. 6:**
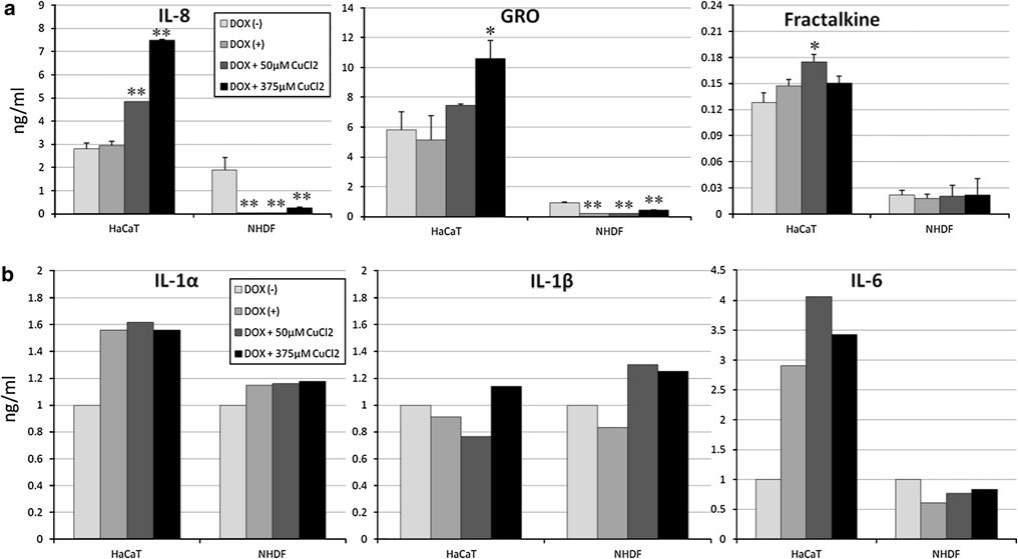
Changes in chemokine and cytokine production in HaCaT and NHDF cells following addition of DOX or DOX + Cu(II) ions to culture medium. **a** Production of chemokines IL-8, GRO, and Fractalkine (ng/ml). HaCaT cells exhibited DOX- and Cu(II)-dependent production of IL-8 and GRO. The values are presented as mean ± SD (*n* = 3). Significantly different from control: *p < 0.05, *p* < 0.01. **b** Production of cytokines IL-1α, IL-1β, and IL-6 (ng/ml). HaCaT cells exhibited DOX/Cu(II)-dependent increased production of all cytokines shown, and NHDF cells exhibited substantially increased production of IL-1β only

In the regions of the rat skin tissue affected by the PEGL-DOX treatment were measured using an antibody-array, markedly increased expression of multiple cytokines was confirmed: the chemokines CINC3 and Fractalkine, the IL-family-inhibitory IL-10, and inflammatory cytokines such as IL-1β and IL-6 (Fig. 4). Therefore, we studied this mechanism in detail in vitro. HaCaT and NHDF cells were treated with various concentrations of DOX (0.1–10 μM) and survival rates were determined after 24 h. Results showed that 1.5 μM exhibited a moderate level of toxicity that was appropriate for toxicity tests to follow (Fig. 1). The presence of the 1.5 μM DOX did not detectably increase the production of chemokines in the HaCaT cells, but the addition of Cu(II) ions caused increased production of the aforementioned CXC chemokines GRO and IL-8, with the production volume dependent on the Cu(II) ion concentration (Fig. 6a). However, in the NHDF cells, neither DOX alone nor the combination of DOX and Cu(II) ions yielded increased production of chemokines; in fact, DOX appeared to inhibit chemokine production.

The effects of DOX and Cu(II) ions on inflammatory cytokine production varied across different cytokines and different cell types (Fig. 6b). HaCaT cells produced IL-1α and IL-6 in response to the presence of DOX, and production was further amplified by the addition of Cu(II) ions. The production of IL-1β rose in the presence of DOX combined with a very high concentration of Cu(II) ions. By contrast, NHDF cells exhibited no noticeable response to DOX alone, and the addition of Cu(II) ions stimulated an increase in only IL-β production.

**Fig. 7:**
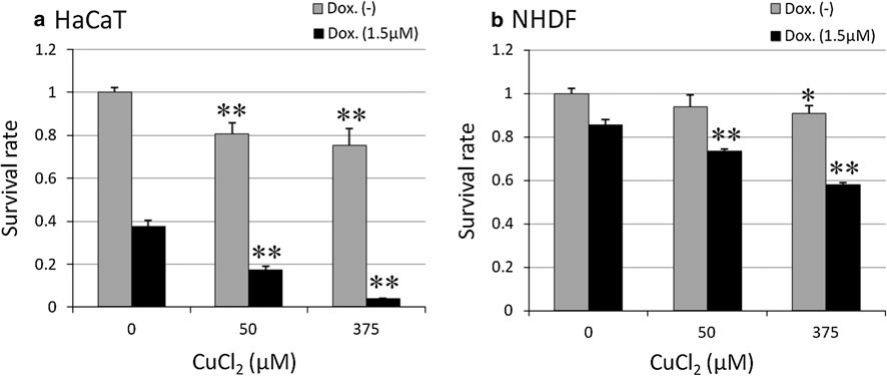
Survival rate of human cells in the presence or absence of 1.5 μM DOX and varying concentrations of Cu(II) ions. **a** HaCaT cells. Survival rate declined with increasing Cu(II) ion concentration in the presence of DOX. **b** NHDF cells showed relatively little response to DOX and Cu(II) ions. The values are presented as mean ± SD (*n* = 3). Significantly different from control: *p < 0.05, **p < 0.01

**Fig. 8:**
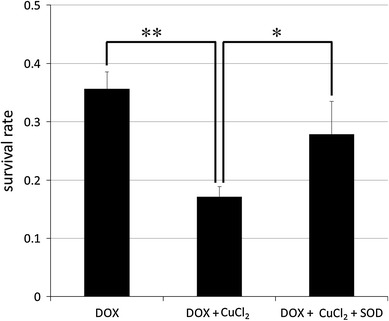
Rescue of DOX/Cu(II)-induced decline in HaCaT cell survival rate by SOD. The values are presented as mean ± SD (*n* = 3). Significantly different from DOX, DOX + CuCl_2_, and DOX + CuCl_2_ + SOD, respectively: *p < 0.05, *p < 0.01

Without the addition of DOX, the survival rate of HaCaT cells remained relatively constant across varying concentrations of Cu(II) ions (Fig. 7). However, in the presence of DOX, the survival rate of the cells rapidly decreased with increasing Cu(II) ion concentration. By contrast, the NDHF cells were relatively unaffected by the combination of DOX and Cu(II) ions. The addition of superoxide dismutase (SOD) improved this HaCaT cell survival (Fig. 8).

## Discussion

A variety of research has been conducted on the relationship between Doxil^®^ (trade name for PEGL-DOX) and HFS, and many important discoveries have already been made. Charrois et al. [7] have analyzed the pharmacokinetics of DOX in rat skin tissue and tumors after multiple doses of Doxil^®^, and results have shown that the half-life of Doxil^®^ is particularly long in the paws. It has become known that multiple doses of anticancer drugs, or possibly a single large dose, can cause an accumulation of cell damage and a speeding up of the cell cycle in keratinocytes, which can ultimately lead to an outbreak of HFS [8]. By using the skin of humans to whom fluorescence-tagged Doxil^®^ had been administered, Martschick et al. [9] have discovered that Doxil^®^ leaks out from the body in the sweat. Both in vivo experiments in mice and rats and in vitro experiments in HaCaT cells led to the conclusion that DOX toxicity in the skin causes hair-loss via denaturation of the sebaceous line [10]. In addition, the idea that Manganese SOD (MnSOD) can suppress apoptosis was introduced in an experiment investigating DOX-induced apoptosis in HaCaT cells [11]. Finally, it has been reported that the coexistence of DOX and Cu(II) generates ROS, which inflict oxidative damage on DNA [12].

As described above, while research on DOX, Doxil^®^, and the skin is plentiful, little is known about why Doxil^®^/PEGL-DOX frequently causes HFS or why the outbreaks occur in the skin tissue. Moreover, there have been no reports of effective treatments for this condition.

The tendency of PEGL-DOX to induce HFS outbreaks in rats where DOX did not led us to the theory that the difference in metabolic stability between the two drugs was the cause of the difference in frequency of HFS outbreaks in humans. In other words, the key difference is that the higher metabolic stability of PEGL-DOX allows it to remain active in the blood for a longer period of time, as illustrated by the presence of PEGL-FS remaining in the rat extremities after 7 h (Fig. 4). This also means an increase in the frequency with which the drug reaches the hands and feet.

In fact, the capillaries are concentrated at the fingertips and the soles of the feet, where the blood flow is high. Unlike three-layer artery or arteriole walls, capillary walls are composed of only a single layer of endothelial cells, which makes capillary walls easy to penetrate with only slight provocation. The capillaries are especially concentrated in the dermis, which suggests that anticancer drugs might easily leak into the dermis and linger there at high concentrations, as indicated by the results of the experiment using the fluorescein drug model PEGL-FS (Fig. 4).

Doxorubicin is known as the most dangerous of all vesicant drugs, which are high-risk drugs capable of causing tissue necrosis upon extravasation [13]. Therefore, the accumulation of DOX in this tissue is extremely cytotoxic.

Many anticancer drugs cause DNA damage in order to induce apoptosis in cancer cells. There have been many experiments that show that apoptosis can be induced by ROS generated directly or indirectly by anticancer drugs [14–18]. It is said that DOX damages DNA by generating ROS and inhibiting Topoisomerase II [12]. Furthermore, it has been reported that the oxidative damage due to ROS was magnified in the presence of Cu(II) ions in experiments using the human promyelocytic leukemia cells [19]. Our own results corroborated the pre-existing evidence that DOX induces apoptosis in keratinocytes via ROS in the presence of Cu(II) ions and that SOD rescues the cells from apoptosis by capturing the ROS in the culture medium [11, 20].

The effects of DOX and Cu(II) ions on cell viability and chemokine production appear to be tightly correlated. The results of our rat tissue staining experiments (Fig. 2) indicate that DOX induces apoptosis in keratinocytes, but not in dermal fibroblasts. Similarly, the results of our cytotoxicity tests (Fig. 7) indicate that, while DOX does not affect fibroblasts, it appears to kill keratinocytes, and it appears to kill at a greater rate in the presence of Cu(II) ions. As for chemokines, the pattern is similar. While fibroblasts do not appear to produce chemokines even in the presence of both DOX and Cu(II) ions, keratinocytes produce the chemokines IL-8, GRO, and Fractalkine in response to DOX, and IL-8 and GRO are produced in a Cu(II) concentration-dependent manner (Fig. 6). The fact that chemokine production spiked in areas of rat skin tissue afflicted with HFS (Fig. 3) suggests that these chemokines represent an important part of the mechanism by which injection of PEGL-DOX leads to HFS.

Yamamoto et al. [20] have reported that the thermoreceptor TRPM2 is expressed on the surface of keratinocytes and plays the role of sensing ROS in the surrounding environment. In response to ROS, these receptors create holes in the cell surface through which Ca^2+^ ions flow into the keratinocytes. The rise of the intracellular Ca^2+^ ion concentration due to this influx induces chemokine production. This mechanism is thought to be responsible for the increase in chemokine production in HFS-affected tissues.

Chemokine production alone is not sufficient to produce the typical HFS state of inflammation. Moreover, it is thought that chemokines do not directly induce keratinocyte apoptosis, but, rather, death factor cytokines are a necessary intermediary. Death factors known to induce apoptosis include TNF-α, Fas ligand, lymphotoxin α, TNF-related apoptosis-inducing ligand (TRAIL)/Apo2 ligand, and Apo3 ligand [21]. Chemokines induce positive chemotaxis in blood cells, which express these death factors. For example, neutrophils expressing Fas ligand migrate to the dermis in response to chemokines produced by keratinocytes. These neutrophils undergo apoptosis in response to ROS, and at the same time caspase-1 is activated inside the neutrophils, and IL-1β is released from the cells [22]. While our fluorescent staining did not show this migration of blood cells (Fig. 2), despite the presumably high level of chemokine production, it is thought that these cells may have undergone apoptosis and been taken up by macrophages, which would explain their absence in the tissue staining photographs.

The prevailing view up until now was that cells undergoing apoptosis do not induce inflammation because they are absorbed by phagocytes or surrounding cells; however, the apoptosis of cells expressing death factors is a different matter. It is known that keratinocytes also express death factors [22], and it is thought that blood cells and keratinocytes undergoing apoptosis due to the presence of ROS cause inflammation by releasing IL-1β. Our results indicate that fibroblasts also produce IL-1β in response to ROS stimulation, and keratinocytes produce IL-1α and ILβ in a manner dependent on Cu(II) ion concentration (Fig. 6). In short, due to the presence of ROS, keratinocytes, blood cells, and fibroblasts produce inflammatory cytokines, which leads to vasodilation, rubefaction, fever, augmented vascular permeability, and swelling; i.e., the HFS disease state.

**Fig. 9:**
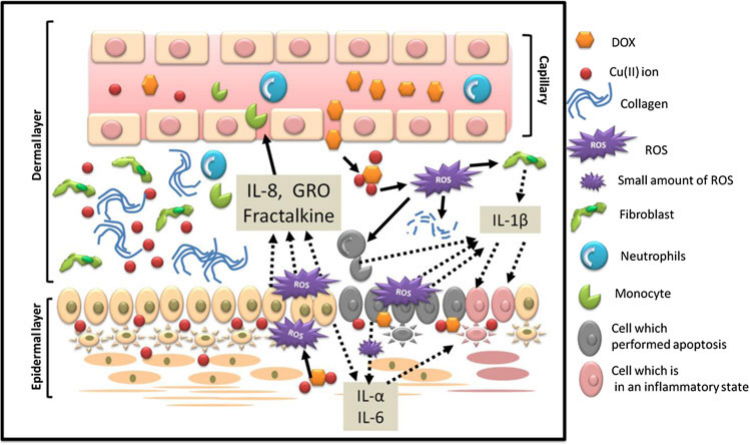
Illustration of HFS mechanism. As known, doxorubicin would produce ROS in the presence of metal ions. The illustration is shown in the case of the presence of Cu(II) ions because doxorubicin would react with Cu(II) ions to produce ROS. Doxorubicin could induce the apoptosis of skin cells by ROS which stimulates keratinocytes (epidermal layer cells) and blood cells by the productions of chemokines and cytokines by themselves. Additionally, ROS cause collagen breakdown

To summarize, the mechanism of HFS development that we propose based on our research is as follows (Fig. 9): (1) DOX penetrates the capillary walls and interacts with Cu(II) ions in the skin to produce ROS; (2) these ROS attack keratinocytes, which release chemokines and the inflammatory cytokines IL-1β, IL-6, and IL-1α; the cytokines induce apoptosis in the keratinocytes, and the chemokines induce positive chemotaxis in blood cells, which in turn release IL-1β and apoptose; (3) fibroblasts release IL-1β in response to the presence of ROS; (4) ROS destroy collagen fibers in addition to causing cell death; and (5) the combination of inflammation and keratinocyte apoptosis due to the accumulated cytokines and collagen destruction in close proximity results in complete skin tissue devastation.

In conclusion, we propose that HFS develops due to the combination three primary factors: (1) the inherently strong cytotoxicity of DOX, (2) the ability of PEGL-DOX to remain in circulation for extended periods of time as a PEG-modified liposome, and (3) the abundance of metal ions in the skin tissue.

However, several factors remain to be investigated, including the influences of different metal ions and the effects of DDSs capable of remaining in circulation for differing periods of time. Having elucidated a detailed mechanism for HFS development, we are now ready to begin the development of drugs to treat or prevent the condition all together. A likely starting point is SOD, which was able to rescue cell viability in the presence of ROS, and therefore implies the potential for a treatment that captures and removes ROS to prevent damage. In the future, we plan to produce even more detailed research on this subject.
